# A guide to direct mechanocatalysis

**DOI:** 10.1039/d1cc05697b

**Published:** 2022-01-13

**Authors:** Suhmi Hwang, Sven Grätz, Lars Borchardt

**Affiliations:** Professur für Anorganische Chemie I, Ruhr-Universität Bochum Universitätsstraße 150 44780 Bochum Germany lars.borchardt@rub.de

## Abstract

Direct mechanocatalysis (DM) describes solvent-free catalytic reactions that are initiated by mechanical forces in mechanochemical reactors such as ball mills. The distinctive feature of DM is that the milling materials, *e.g.* the milling balls themselves are the catalyst of the reaction. In this article we follow the historical evolution of this novel concept and give a guide to this emerging, powerful synthesis tool. Within this perspective we seek to highlight the impact of the relevant milling parameters, the nature of the catalyst and potential additives, the scope of reactions that are currently accessible by this method, and the thus far raised hypotheses on the underlying mechanisms of direct mechanochemical transformations.

## Abstract 2

Direct mechanocatalysis (DM) describes catalytic reactions in mechanochemical reactors such as ball mills with the distinctive feature that the milling equipment itself is the catalyst.

## Graphical abstract

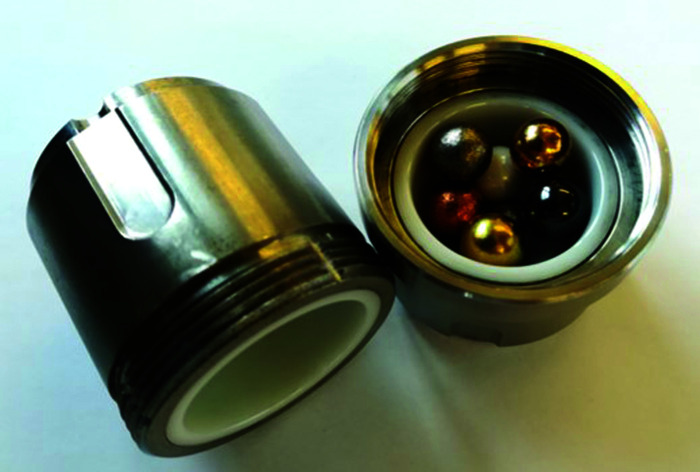

## Introduction

Mechanochemistry (MC) refers to chemical reactions which employ mechanical energy as a driving force.^1^ The mechanical energy can be provided for instance by using pestle and mortar, by the collision of milling balls inside ball mills, or by sheering forces in extruders. In ball mills, the nowadays most frequently used method, the collision of milling balls transfers energy to the reactants. MC is an inherently solvent-free concept, wherein solids can be reacted without the need of dissolution. This does not only increase the reaction's sustainability, as solvents are one of the major contributors to waste accumulation, it also increases the reaction's energy-efficiency as it is no longer necessary to expend energy to heat, pump, or cool a reaction component that is not a part of the final product. Moreover, if the substrate's solubility in an appropriate medium is no longer required, the list of possible reactants and reactions increases greatly. While mechanochemistry can make use of these major advantages if applied in solid-state chemistry, mechanochemical methods can technically cover various reactant states from gas to liquid to solid.^2^

Mechanochemistry is accordingly gaining substantial attention with the growing needs for greener approaches in synthetic chemistry. MC is applied in a variety of chemical disciplines with examples ranging from cocrystals,^3^ metal–organic frameworks,^4^ molecular rearrangement,^5–7^ materials chemistry,^8^ polymer chemistry,^9^ porous materials,^10^ organic chemistry,^11^ biochemistry,^12^ pharmaceuticals,^13^ API (active pharmaceutical ingredient)^14,15^ to energy storage materials.^16^

One of the key subjects in the context of green chemistry and one of the major breakthroughs in chemistry in general is catalysis. It increases the energy economy during the reaction, thereby enabling faster and more selective reaction pathways to create sought-after molecules. The combination of mechanochemistry with catalysis is thus an obvious win–win situation.^17^ Indeed, a plethora of catalytic reactions have already been performed in ball mills. Well-known catalytic reactions such as, C–N and C–C cross coupling,^18,19^ cycloaddition,^20^ polymer reactions,^21^ C–H activation^22^ were successfully conducted in the ball-mill, although the question whether they process *via* comparable reaction mechanism or entirely different remains widely unanswered. Interestingly, some of these reactions that were conducted mechanochemically showed remarkable advantages over the corresponding conventional methods. For instance, in 2008, the Ondruschka group demonstrated Suzuki-type C–C cross couplings under solvent-free conditions in a planetary ball mill.^19^ In this report, high yields were achieved for various couplings of aryl halides with phenylboronic acid in short reaction times while being more sustainable with respect to comparable conventional reaction conditions. In 2011, the same group reported a copper-catalysed alkyne–azide-click cycloaddition reaction in a ball mill.^20^ This reaction was conducted under solvent- and ligand-free conditions, and produced the product in high yield in a short reaction time of 10 minutes. Another interesting example is the highly stereo-selective mechanochemical reaction of aziridine containing polymers with dipolarophiles reported by H. J. Yoon group in 2020.^21^ This reaction was shown to not proceed *via* conventional reaction pathways, thus underscoring the promise of employing mechanochemistry to pioneer new synthetic approaches.

However, certain disadvantages cannot be overcome with these approaches. As a typical feature of the utilized homogeneous catalysts, their separation and recycling remains challenging.^23^ Although some reactions have been conducted with simple metal salts, other still require elaborate ligand systems. Those ligands are often costly and involve multiple synthetic steps to prepare.

A major breakthrough to overcome these downsides employs a concept called “direct mechanocatalysis” (DM). This concept is characterized by combining the functional aspects of energy input and catalysis in one single component. To achieve this, the milling ball is manufactured out of the catalytically active metal or its alloy and catalyses the reaction while accelerating and colliding inside a milling jar. This approach is at a first glance counterintuitive, since conventional catalysis relies on either the maximisation of catalytically active surface (heterogenous catalysis) or the high dispersion of the catalyst in the reaction mixture (homogenous catalysis). In DM, the catalyst is a milling ball, *i.e.* a sphere, thus that geometrical object with the smallest possible surface for a given volume. Moreover, it is clearly in a different phase than the reactants. DM is thus conceptually different from the two conventional types of heterogeneous and homogeneous catalysis. However, the vibration and consequently the collisions between balls or between balls and the vessel are capable of refreshing the surface area of the ball several times a second and thus creating an extensive apparent surface area for catalysis. Moreover, due to the mechanisms of mechanochemistry, the catalyst is at all time in precisely the right position namely in the point of the collision. Both of these factors have led to the development of several reactions using this concept, namely: cycloaddition reaction,^24^ C–C coupling type reactions^25,26^ and hydrogenation reactions.^27,28^ The application of direct mechanocatalysis in these examples has several advantages such as:

1. The separation of the catalyst from the reaction mixture is as easy as separating the powder from the balls.

2. The reusability of the catalyst is easy and typically does not require additional steps.

**Fig. 1:**
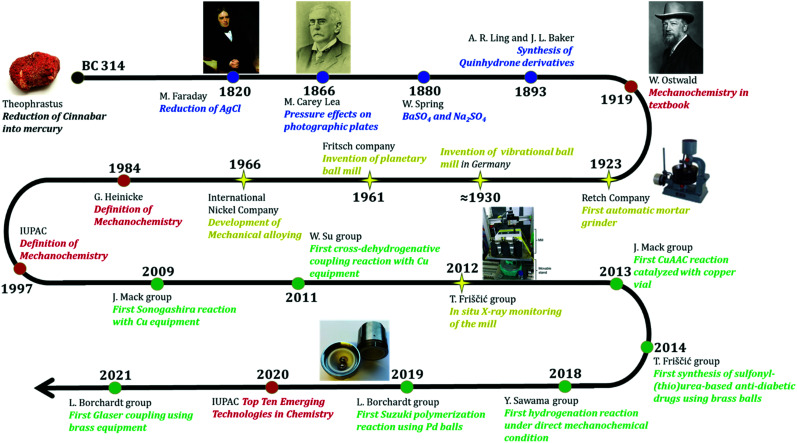
Timeline of mechanochemistry and “direct mechanocatalysis”. Blue: pre-modern time; red: important milestones of official recognition for mechanochemistry; yellow: major technological development; green: first reports of specific direct mechanocatalytic reactions.

Since the concept is a rather new addition to the toolbox of mechanochemists and organic chemists alike, this article will discuss crucial aspects in conducting chemical reactions by DM which are: (1) the impact of classical milling parameters, (2) balls, mills and the design of milling ball as catalyst, (3) possible reactions and necessary additives, and (4) hypotheses and so far known details on the catalytic mechanism. But let's briefly take a look of the historical development to establish a frame of reference (Fig. 1).

## History from mechanochemistry to direct mechanocatalysis

The often discussed and first documented example of mechanochemistry was reported by Theophrastus of Eresos in 314 B.C. in his book “On Stones”.^29^ Herein, cinnabar was pestled in a copper mortar bar with vinegar to yield mercury. Not only it is considered to be a first recorded process for obtaining a pure metal from a compound, it already showed that the milling equipment itself, *i.e.* the mortar, can get involved into a reaction – a detail of major importance for the development of DM. Afterwards not much has been documented, but from contemporary art, we know that the mortar continued to be a main stay in alchemist's and early chemists’ laboratories until it fell out of favour with the construction of elaborate glassware. During the pre-modern time, mechanochemistry started to be more intensely and systemically investigated and documented. In 1820, Michael Faraday found a mechanochemical interaction between zinc and silver chloride.^30^ He was originally investigating if hydrogen is part of the reduction process of silver chloride. Instead, he found that trituration of dry zinc and silver chloride produces silver. Matthew Carey Lea is the first known chemist who systemically investigated the chemical effects of mechanical actions.^31^ His first mechanochemical observation was in 1866, where he considered a pressure effect on sensitized photographic plate. He used a glass rod to put a pressure on a photographic plate and was able to develop an image that resembled the image produced by light. Walthère Spring is another prominent mechanochemist in the nineteenth century who intensely investigated chemical effects of mechanochemical actions along with M. Carey Lea. One of his well-known experiment is salt metathesis reaction between barium sulfate and sodium carbonate conducted in 1880.^32^ The product was obtained by repeated compression and pulverization. According to F. Garcia's review,^33^ this is an early example that poor solubility of reactants – in this case barium sulfate – can be overcome with mechanochemical approach by simply grinding the reactants of solid states.

In 1893, Ling and Baker could prepare halogen derivatives of quinhydrone, by grinding the reactants using pestle and mortar.^34^ Interestingly, their methods also include adding small amount of liquid such as water or light petroleum to the grinding system, which is nowadays referred to as “liquid assistance grinding”. In 1919, the term “mechanochemistry” was officially coined in the “Textbook of General Chemistry” by Wilhelm Ostwald.^35^ According to L. Takacs, Ostwald was inspired by M. Carey Lea's experiment of the AgCl and HgCl_2_ decomposition upon trituration, which was not observed with thermal conditions.^36,37^ Since his classification, mechanochemistry has been considered as a branch of chemistry along with thermochemistry, photochemistry and electrochemistry. The more detailed and widely used definition of mechanochemistry was established later in 1984 by G. Heinicke.^38^

During the twentieth century, there were important inventions that later directly affect the advance in mechanochemistry. Major examples are the first automatic grinder by the Retsch company in 1923, the invention of vibrational ball mills in Germany in 1930 and the invention of planetary ball mills by the Fritsch company in 1961. The development of new mills has mainly been conducted for the mining industry and to facilitate chemical analysis. In the last decade, however, new mills have been developed with mechanochemistry in mind. The detailed historical development of the technical aspect has been well reviewed previously by C. Suryanarayana.^39^ We would like to direct the readers there for more detailed information. With new technologies and a growing necessity of greener and more sustainable methods in chemistry, the research in mechanochemistry has gained traction with the beginning of the 21st century. More and more reactions were adapted and it was the J. Mack group in 2009 who were the first to report the application of catalytically active milling materials. They utilized a copper vessel as a co-catalyst in the Sonogashira reaction and were able to completely replace the commonly used copper(i) iodide.^25^ Afterwards W. Su and co-workers established a cross-dehydrogenative coupling reaction by employing copper milling balls as catalysts.^40^ Consequently, several other reactions using copper and copper alloy milling balls and vessels have been reported.^10,24,41,42^

But this approach is by no means limited to copper. In 2018 the Y. Sawama group reported that a cascade of reactions can be catalysed by steel. They observed that the chromium content in steel can catalyse a water-splitting inside a planetary ball-mill and that the released hydrogen can consequently be activated by the nickel content to conduct hydration reactions.^27^

Nickel itself has also been used in the form of pellets to catalyse the synthesis of substituted cyclooctatetraene by a [2+2+2+2] cycloaddition by the Mack group.^24^

Lately our group has been working on expanding this concept further by utilizing palladium milling balls for cross coupling reactions. While doing this we have experienced many problems and gained deeper insight into the workings of direct mechanocatalysis that we want to share in the following chapters.

## Guide to direct mechanocatalysis – part 1: milling parameters

Mechanochemical reactions are governed by parameters, which are of different nature with respect to traditional solution-based chemistry. For instance, filling degree, milling speed, and vessel material play significant role in mechanochemical reactions. Many of the lessons learned here can be directly applied to DM, while others have to be revised. Throughout human history, mortar and pestle have been the most commonly used mechanochemical reaction tools.^1^ However, since this method is conducted manually, it results in a lack of reproducibility and low efficiency due to the low energy input. The reaction parameters of automated ball mills are much more defined, directly controllable, tuneable and foremost reproducible.

The use of ball mills grants control over the exact milling time and allows minute control over the energy input throughout the milling process. Despite the various choices of mechanochemical equipment, the most commonly used types of mills on a laboratory scale are vibratory ball mills and planetary ball mills.

The reaction time, or milling time, is one of the most intuitive reaction parameters, typically 0.5–4 hours for DM. In mechanochemistry, a longer reaction time leads to more productive collisions and thus is influencing reactions in the same way as in classical chemistry. For instance, the cyclisation of substituted alkynes to generate substituted cyclooctatetraenes catalysed by nickel pellets as milling material (Table 1) was shown to yield 54% of the product after 1 h and 94% after 16 h.^24^ One notable aspect however, is the continuous transfer of mechanical stress onto the reactants. This process may lead to unwanted degradation of the desired product if a too long milling time is chosen. In fact, during our investigation of the direct mechanocatalytic synthesis of poly *para*-phenylene, we first prepared the polymer *via* a palladium catalysed conversion of 4-bromophenylboronic acid (Table 1). Herein, the highest degree of polymerization, *i.e.* the highest average length of the polymer chains was obtained after 4 h. Longer milling times resulted in shorter average chain length, an observation that we attributed to the mechanical stress-induced destruction of the polymer.^22^

**Table:** Influence of the milling time on the yield of DM reactions^24^ and the development of the degree of polymerization (DP) over time^26^

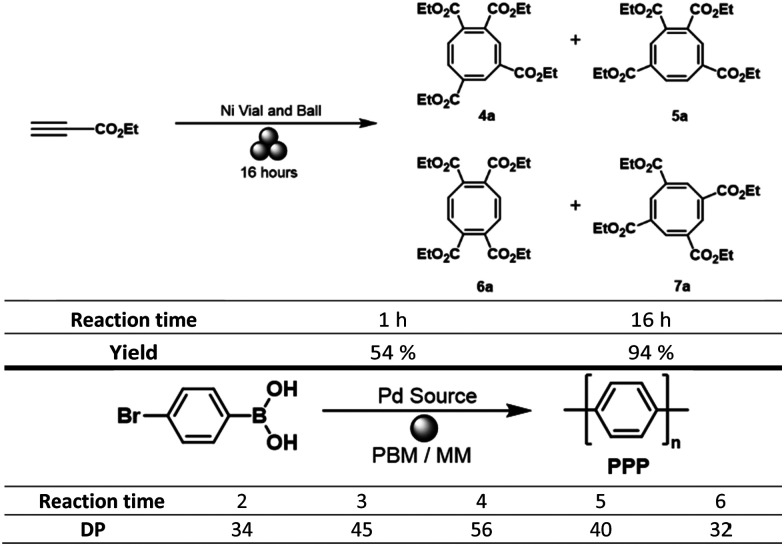

Furthermore, the milling process also causes abrasion of the milling equipment and prolonged milling thus leads to unnecessary contamination of the reaction mixture, as well as the slow wearing down of the milling equipment. As such in mechanochemistry, one often has to fine-tune the reaction time to minimize adverse effects while maximizing the yield of a reaction. This holds true for direct mechanocatalysis as well. Our group observed abrasions of brass balls under the Glaser coupling in ZrO_2_ vessel.^43^ The amount of abraded brass balls (0.7–2.3–8.9 mg) increased as the milling time increased (20–90–180 min).

The milling frequency/milling speed is the most commonly modified parameter when conducting reactions in ball mills and it usually varies between 20–35 Hz or 400–800 rpm. This parameter is of great significance due to its direct influence on the energy input on the reactants and has no direct equivalent in solution-based reactions. It cannot be directly compared to the speed of a magnetic stirrer since the milling frequency accounts for both the better dispersion and the reactive collision. In general, higher frequencies thus lead to faster reactions. This is not only caused by the number of collisions but also by the transferred energy. For higher frequencies the acceleration and in turn the impact energy of the milling balls is higher than for lower speeds. For instance, the approximate kinetic energy of a milling ball (Table 2) at 30 Hz was investigated to be 1.958 mJ, which is higher than as four times as energy of the milling ball at 15 Hz.^44^ This again holds true for DM and is enhanced by the fact, that it also causes a higher number of interactions between catalyst and reactants. A 10 minutes Suzuki reaction (Table 2) conducted at 20 Hz yields only traces, at 25 Hz 31%, and at 35 Hz 85% of product. On the other hand, higher milling frequencies are accompanied by an increase of thermally dissipated energy, which leads to a temperature increase inside the vessel. The topic of temperature will be discussed separately later on. Furthermore, higher milling frequencies increase the stress onto the milling balls and as such lead to an increased abrasion during the reaction.

Approximate kinetic energy of an ideal impact of a 1.43 g milling ball at different frequencies.^44^ And variation of yield of a Suzuki reaction between iodophenyl and phenylboronic acid with one palladium milling ball for 10 minutes in a MM500

**Table:** 

Frequency (Hz)	15	20	25	30
Kinetic energy (mJ)	0.489	0.870	1.359	1.958

**Table:** 

Frequency (Hz)	20	25	35
Yield (%)	Traces	31%	85%

The filling degree describes the volume ratio of either the milling material (milling balls) or the grinding material (reactants) to the volume of the milling vessel. This, again, has no direct comparison in solution-based chemistry but heavily impacts mechanochemical reactions. In classic mechanochemistry, a filling degree of *ca.* 30% of milling balls relative to the vessel volume is generally believed to be the optimum.^45^ While this rule stems from the producers of ball mills, an in-depth study by A. Stolle and co-workers identified that it is rather around a filling degree of 40% where mechanochemical reactions proceed the best. According to the A. Kwade group, no detachment of balls was observed with this ratio and balls are always in contact with each other or the wall of the vessel, which result in excellent efficiency of compression and shear stress. This rule mainly applies to planetary ball mills.

Direct mechanocatalytic reactions, however, are mainly conducted in vibration ball mills and only 1–2 balls are employed.^45^ Here, the amount of powder can be miniscule and the filling degree is most commonly not considered as such. To achieve a specific desired filling degree, either the appropriate amount of substrate or supplementation with an inert additive such as NaCl can be employed.^33^ This is important for avoiding abrasion since ball–ball or ball–vessel collisions are not contributing to the reaction if no powder is present on the impact spot. In DM, the filling degree has up to now not been investigated systematically, however, we propose that it can be interpreted similar to the catalyst concentration in classical solution-based chemistry. It influences the ball to powder ratio and thereby effects the energy dissipation and transfer in the vessel during the reaction. Assuming a constant number of milling balls, the filling degree dictates the average free path length of the milling material and thereby its energy transfer properties.

**Fig. 2:**
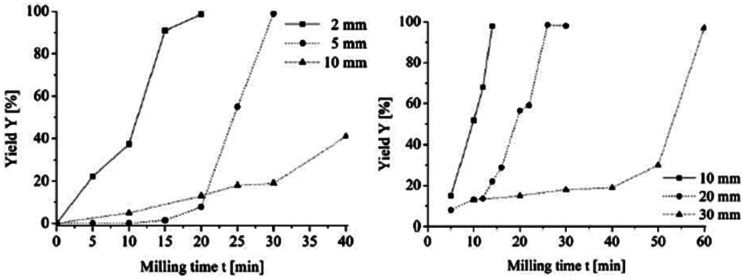
Influence of milling time, *t*_reaction_, and milling ball diameter, *d*_MB_, on the yield of the Knoevenagel condensation of vanillin and barbituric acid (equimolar ratio). Left: PBM Fritsch P7 premium line (*V*_MV_ = 0.045 L, *n* = 20 mmol, rpm = 800 min^−1^). Right: PBM Fritsch P6 (*V*_MV_ = 0.25 L, *n* = 100 mmol, rpm = 650 min^−1^).^46^

The size of the milling balls has a powerful effect on the impact energy. Along with the frequency, this parameter is commonly adjusted with the goal of modifying the energy input into the reaction (Fig. 2).^46^ The most commonly employed size of milling balls is 5–15 mm. Principally, bigger milling balls carry higher kinetic energy per individual ball and can thus transfer higher amounts of energy onto the reactants. For instance, Boldyreva and Michalchuk groups investigated the effect of mass of milling balls on the polymorphic conversion of anhydrous caffeine.^47^ According to the literature, larger balls showed faster conversion rates due to their higher impact energies and higher surface areas. By contrast, small balls induce more frictional action, which can be useful when the goal is to achieve efficient mixing instead of high energy input.^45^ A downside of smaller balls is their tendency to get attached to each other when reactants melt or become sticky, which may hinder the effective milling motions. If these issues are encountered, employing bigger balls can be an attractive solution. Additionally, the desired particle size can also be controlled by adjusting the size of balls. For instance, the expected grain size gets smaller when smaller balls are used. It is commonly thought that the final grain size is about 1/1000 of the ball size, though this also depends on other factors such as the filling degree. Interestingly, in some reactions such as the dehydrogenation of γ-terpinene, the size of the milling balls was shown to not have an observable influence on the conversion and chemical yield while adjustment of the milling frequency and the number of milling balls was shown to notably impact the outcome of the reaction.^48^ Boldyreva group has investigated the influence of size, mass and material of the milling balls in a cocrystallization reaction between theophylline and nicotinamide wherein the material of the balls was found to be particularly impactful.^49^

In the context of DM, the size and number of milling balls directly influences the available catalytic surface area on which the chemical reaction can take place. Let's consider the following analogy to traditional heterogenous catalysis: assuming that in a given reaction the total mass of utilized milling balls is kept constant (as it is typically the practice when conducting mechanochemical reactions), then a reduction in the size of the milling balls size implies to the use of a higher number of balls and thereby an increase in catalytically active surface area. *A priori*, this would suggest an increase in conversion rate when smaller balls are used. However, it is important to recognize that the surface of the milling material is not static but rather dynamic, as mentioned at the beginning of this chapter, due to the constant collisions and the grinding motion of the balls. As mentioned above, smaller balls result in smaller energy input, therefore it should be considered whether higher energy input or more frequent interactions of the catalytic balls and the reactants is more critical for reactions. Unfortunately, to the best of our knowledge, no systematic study on ball size that investigates the correlation between static and dynamic catalytic surface area and their effect on the conversion rate of a reaction has been conducted for DM. Most commonly either one catalytically active milling ball was used or the ball size was not varied at all. In our work we usually employ one or two 10 mm palladium milling balls while the Y. Sawama group is utilizing 5 mm steel balls in their reactions. Up to now, these ball choices are mainly governed by availability and price. While milling balls made from ceramics or steel are readily available because of their abundance in other applications like ball bearings, noble metal balls have to be hand-casted. Additionally, our investigations show in the use of two or more milling balls lead to a higher amount of abrasion.

**Fig. 3:**
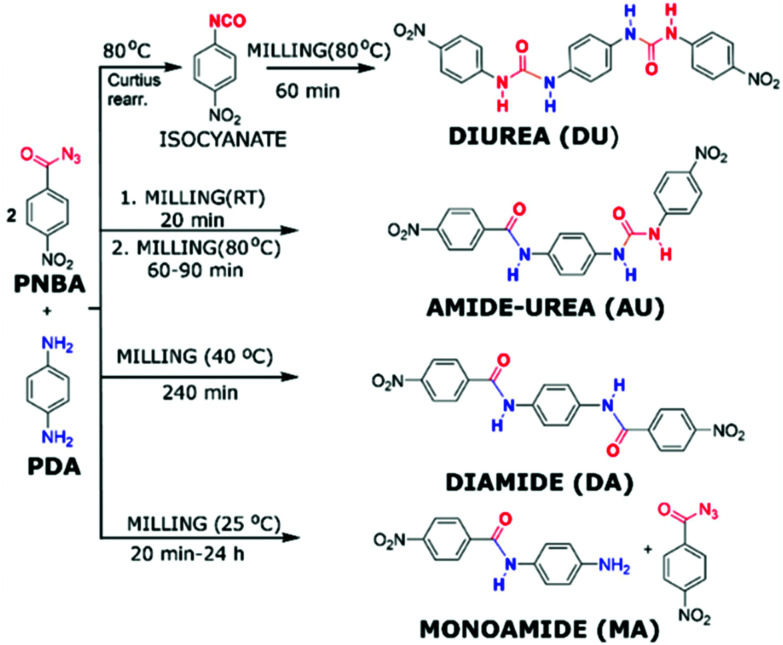
Reaction between PDA and PNBA at different temperatures investigated by Užarević group.^50^

The reaction temperature is the parameter which is most controversially discussed in mechanochemistry. In traditional solution-based reactions it is well understood and easily controlled *via* an external heating bath. Additionally, the presence of a continuously stirred solvent allows for the fast and even transfer of heat in such reactions. There are two general approaches: (i) conduction of a reaction at higher temperatures to increase the kinetics and (ii) conduction of a reaction at lowers temperatures to obtain kinetic reaction control. Unfortunately, controlling or even just measuring the temperature of a mechanochemical reaction is less intuitive than in the case of solution-based reactions. Attempts to control the milling temperature include efforts by Užarević group (Fig. 3), who demonstrated that the major chemical product of the reaction between *para*-phenylenediamine (PDA) and *para*-nitrobenzoyl azide (PNBA) could be altered through control of the reaction temperature using a water-heated milling reactor.^50^ If the reaction was run at elevated temperatures such as 80 °C, the resulting product featured urea-linkages while at lower temperatures, the generation of connecting amide bonds predominated. These results emphasized the potential of controlling the vessel temperature with the goal of directing the mechanochemical reaction towards the desired product. In contrast to the heating of the reaction vessel, the use of coolants such as liquid nitrogen during mechanochemical reactions is referred to as “cryo milling”.^39^ However, these conditions are not well suited for organic synthesis in the ball mill and few reports for the use of cryo milling in organic synthesis are available. Within the arena of low-temperature mechanochemistry, the group of J. Mack have recently shown that lowering the temperature during the mechanochemical reduction of 4-*tert*-butylcyclohexanone with sodium borohydride provided good diastereomeric selectivity for the resulting alcohol product, which also exceeded the selectivity under comparable solution-based conditions.^51^ These results amongst other experiments, however, question the idea of mechanochemical reactions as a whole, or as J. Mack once so poetically highlighted this as “shaking *vs.* baking”. The question arose whether the main driver behind mechanochemical reactions is the introduction of mechanical force, *i.e.* shaking, or if the milling balls “only” mix the reactants and transfer thermal energy which then initiates a classical thermal reaction. There is interesting results from G. Kaupp group that conducted different condensation reactions using carbonyl compounds by ball milling, kneading and melting, wherein they identified the importance of the reaction temperature to achieve satisfactory conversions.^52^ Direct mechanocatalysis can help to answer this because the reaction can only occur on the catalytically active milling ball and not in the reaction media. A controversially discussed idea is the “Hot-Spot-Theory”, wherein mechanochemical reactivity is explained by high temperatures of several thousand degrees existing for a fraction of a second. However, if a reaction can be influenced simply by increasing the vessel temperature to 100 °C, then the idea behind the Hot-Spot-Theory becomes illogical.††The role of temperature in mechanochemical reactions is way too complex to be discussed in a sufficient manner in this contribution. The whole topic deserves a more in-depth presentation and evaluation of arguments for all sides. We thus encourage the reader to read the work of James Mack, and Stuart L. James on the topic.^51,62^ We would like to highlight F. Delogu group, which has reported several investigations on this topic,^53,54^ for instance, they discussed the influence of temperature on the mechanical alloying of Cu–Nb powder mixtures.^55^ At room temperature, mechanical treatment of Cu–Nb powder mixtures led to the formation of homogeneous amorphous phase, on the other hand, nanostructured Cu–Nb composites were observed above 650 K. In direct mechanocatalysis, the role of temperature remains to be investigated. Up to now, the publications have mainly focussed on highlighting new protocols or establishing a basic understanding of the underlying principles. It will be interesting to investigate if the role of temperature remains the same for direct mechanocatalysis or if the reaction is limited purely by the number of reactive collisions.

## Guide to direct mechanocatalysis – part 2: the catalyst

In mechanochemistry, the milling equipment is usually carefully chosen with chemical and mechanical resistance as well as density in mind. Unfortunately, conventional milling materials such as steel, ZrO_2_, SiO_2_ and WC are not catalytically active in most chemical reactions (Table 3). In DM, the milling material has to combine classical requirements with the desired catalytic capability. However, catalytically active metals such as Cu, Ni, Pt and Au are often soft in their pure form and thus do not meet the mechanical requirements for milling materials. This problem becomes evident from all early work on DM, where abrasion was investigated.^24^ In our research on the Suzuki polymerisation, we observed a steady weight loss of the palladium ball.^26^ Similar observations were made for copper-catalysed Glaser coupling, where abrasion in the range of 100 mg or more per ball and hour occurred.^43^ This observation has become one major point of critique of DM. Several key claims in the theory of DM are hardly defendable when large fraction of finely dispersed catalytically active powder is abraded into the reaction mixture. In order to proof whether DM reactions are happening on the milling ball surface or in the bulk powder, several reference experiments are made. In our study of the Glaser coupling we could demonstrate that copper powder alone is not catalysing the reaction, while copper balls are perfectly capable catalysts. Furthermore, the presence of intermediate copper-acetylides was not detected in the bulk solid, but could be shown on the surface of a milling ball by Raman spectroscopy.^43^ Similar observation was made for the Suzuki reaction with palladium powder. Nevertheless, abrasion diminishes the benefits of direct mechanocatalysis as a whole and needs to be minimized or preferably eliminated altogether.

**Table:** Typical milling materials and their application in DM

Material	Hardness (Moh's)	Known reactions	Comments
WC	9.0	N/A	Corroded by strong acids
			Not resistant to oxidizers
ZrO_2_	>9.0	N/A	Resistant to oxidizers
			Resistant to strong acid and bases
Au	2.5	C–C couplings	Resistant to oxidizers
		Epoxidation	Resistant to most acids
		Hydrogenation	Allows for anchoring of thiols and phosphines
Cu	3.0	Glaser coupling	Corroded by strong acids
		Sonogashira	Not resistant to oxidizers
Pd	5.0	C–C couplings	Corroded by strong acids
		Hydrogenation	Resistant to oxidizers

**Fig. 4:**
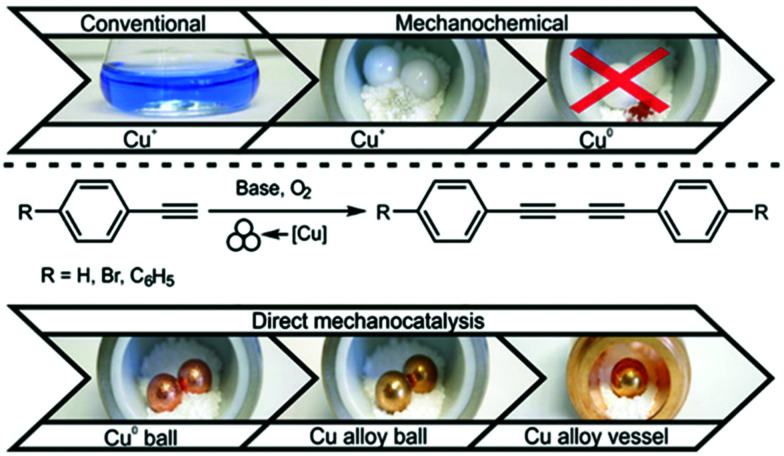
Tuning of milling material for enhanced yield under direct mechanochemical Glaser coupling of phenylacetylenes.^43^

To avoid abrasion in the first place, one has to ensure that the catalytically active part of the milling equipment is the hardest part of the setup. In fact, when there is a difference in hardness between the utilized balls and the vessel, the harder material will grind out the softer material which leads to severe abrasion and potential damage to the equipment. In the exploration of the Suzuki reaction we utilized the latter approach. In order to investigate the reactions *in situ* a transparent vessel material is needed and we turned to perfluoroalkoxy alkane (PFA) for the job. We noticed that the utilization of PFA milling vessel reduced the abrasion of palladium from 113 mg as observed in a ZrO_2_ vessel to below 1 mg. Another interesting method to solve these problems is the utilization of alloys as milling materials, which results in enhanced hardness of the equipment while still having the desired properties. For instance, Friščić group used a brass ball in a stainless jar in the mechanochemical synthesis of the anti-diabetic drug tolbutamide.^41^ Herein, the utilized milling ball allowed for a direct mechanocatalytic reaction using copper-catalysis. Notably, the brass ball did not only compensate for the undesirable softness of elemental copper but also show superior catalytic performance. In our study of the Glaser coupling the abrasion could be reduced from over 300 mg per reaction to 3–5 mg by utilizing either brass or bronze milling balls. Additionally, we could also observe higher yields for the alloyed milling materials. At first glance, this is a counterintuitive observation since the catalytically active metal surface is reduced by utilizing alloy. However, the harder milling materials can be more efficient in transferring energy onto the reactants and the more finely dispersed active metal centres on the ball surface might ease accessibility for the reactants. Another factor might be the absence of abraded metal powder which might hinder energy transport. This indicates that the active surface area in the alloyed milling balls is not decreased. If the vessel and ball material is fine-tuned, the results can even be enhanced.^43^ Employing a bronze ball in a bronze vessel showed almost no abrasion due to the better match of hardness between ball and vessel (Fig. 4). An optimized degree of abrasion of vessel and balls was obtained with an aluminium-bronze vessel that showed the second highest yield of 67% with only 1.0 mg of abrasion. Furthermore, if the desired metal catalyst is utilized as an alloy material for the balls, the price economy should be considered. For instance, unlike affordable zirconium dioxide or brass balls, expensive Pd balls could be financially burdening to replace regularly. In this case, using a vessel of a softer material can be cost-efficient.

Up to now, only metal catalysts have been employed in DM reaction, since they are easy to shape in the desired form of balls and vessels. The area of catalysis is, however, not limited to metals and especially in industrial applications, acid catalysis plays a major role. This whole field has not been transferred into DM up to now. However, we observed that silica nanoparticles influence certain mechanochemical reactions, thus agate vessels are a promising field of research for the future. Moreover, shaping of solid catalysts into balls should be considered. Zeolites are commonly shaped into pellets and might be applicable in combination with softer vessel materials. The vessel or milling ball itself might also easily be modified to enable the anchoring of organic groups on the vessel surface. This would enable a plethora of organocatalytic reactions if they can withstand the rather harsh mechanical conditions.

## Guide to direct mechanocatalysis – part 3: the additives

Most people think of solid–solid reactions in the context of mechanochemistry, but in fact many reagents employed are liquid and even gas–solid reactions have been reported. If gases are employed, they can be used to pressurize the milling vessel themselves or be *in vitro* generated by other means. J. G. Hernandez and co-workers used glass ampules, which would break upon starting of the mill, to introduce gases inside the ball mill.^56^ For DM all types, solid–solid,^41^ solid–liquid,^57^ and solid–gas^27^ have been investigated. While in our group the first two are done exclusively, the Y. Sawama group has utilized the generated hydrogen to conduct hydrogenation reactions using steel vessels. In the Glaser reaction investigated by us, the oxygen inside the milling vessel was required for the reaction and its absence quenched the reaction. In general, inert conditions are not strictly required for many DM reactions as we have shown for the Suzuki coupling reaction. The absence of advanced ligand systems in DM reactions offers even more freedom with respect to reaction atmosphere.

In terms of non-reactive additives there are two classes commonly utilized in mechanochemistry: Liquids for the so-called liquid assisted grinding (LAG) and solids as bulking agents. In DM, there is a third potential type of additive – ligands.

**Fig. 5:**
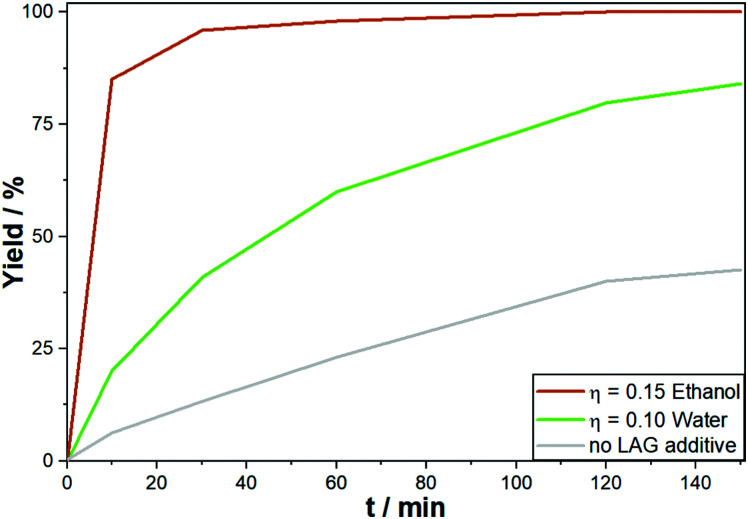
Development of yield over time for the DM Suzuki reaction between phenyliodide and phenylboronic acid with and without LAG conditions.

Although mechanochemical reactions are most often conducted in a pure solid state, the addition of controlled volumes of liquids can be used to influence the reaction. This LAG approach can alter the product selectivity, enhance the conversion rate or enable a reaction altogether. By definition, the amount of the liquid does not exceed 2 μL liquid per 1 mg powder (*η* = 2) and as such is insufficient to dissolve the reactants entirely. While the precise mechanism of LAG is not understood up to now, possible positive effects might be linked to an increased wettability of the surface and/or an increased diffusion rate of the reactants. In direct mechanocatalysis, it was the LAG approach alone which reduced the reaction times from 6 hours down to only 10 minutes (Fig. 5) if either water or ethanol is employed in an *η* = 0.15. One major challenge is that for each reaction type the optimal LAG conditions can vary and screening is mandatory. For example, in the synthesis of porous polymers, water was quenching the reaction while chlorinated solvents or diethyl ether were beneficial, both of which have no effect on the Suzuki reaction.^58,59^

As outlined above, it is important to determine the ideal solid filling ratio to facilitate an optimized energy transfer. If the amount of powder it too little, the collision of milling balls might not result in an effective energy transfer to the reactant, but rather cause high abrasion on the milling material. Similar problems can occur, for example, if the reactants clump up in the course of a reaction caused by using liquid reactants or the deliquescence of hygroscopic substances. Adding a defined amount of inert bulking material to a reaction greatly improves these issues by making the mixtures more neat and homogenous.^60^ Supplemental grinding materials can also act as buffer between balls during the milling thus avoiding abrasion. Alkaline carbonates and chlorides are the most common choice of inert solids without detrimental hygroscopic properties and with reasonably high melting points. Other choices involve silica gel or sand, which have to be considered careful due to their abrasive nature. In our study of the Suzuki reaction, we usually utilize an excess of the base, potassium carbonate, as bulking material. It can be easily separated during the workup and is cheap, secure and environmentally benign. Apart from the role as a grinding supporting material, additives may also be chosen for the reaction itself. For some catalytic reactions, the use of surfactants or ligands may appear interesting. For direct mechanocatalysis in particular, we have found them to be detrimental for the reaction. For instance in our investigation on the Suzuki polymerisation reactions, the addition of common ligands like triphenylphosphine or 1,5-cyclooctadiene quenched the reaction completely.^26^ In this case it is thought that the ligands bind to the metal surface of the milling ball and thereby block the catalytically active area for the Suzuki reaction. However, this observation may be specific to the investigated system. Another interesting additive we utilized in our research is potassium iodide. In our experiments we could show an *in situ* interhalogenation reaction enhancing the reactivity of bromide-substrates in the Suzuki coupling.

## Guide to direct mechanocatalysis – part 4: the mechanism

**Fig. 6:**
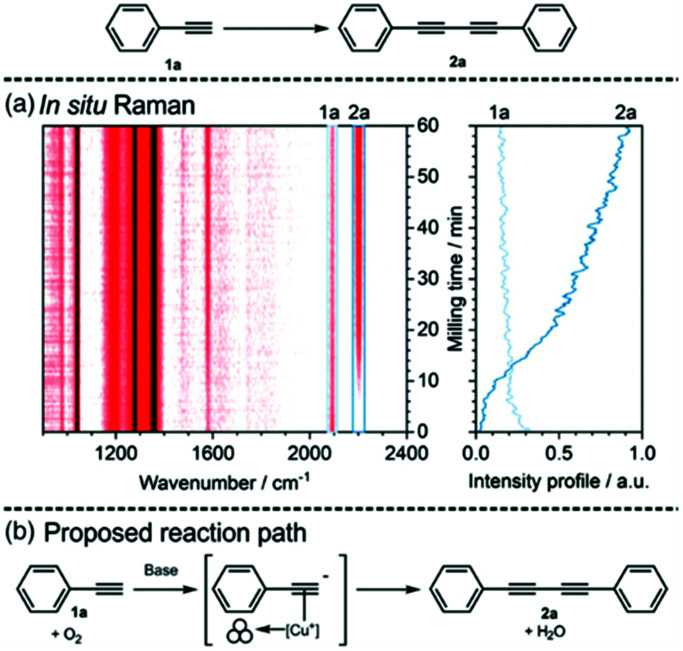
Proposed reaction path of Glaser coupling through copper catalyzed DM (a) *in situ* Raman spectrum (b) scheme of proposed reaction path.^43^

The first question that has to be answered in this context is whether the catalysis is taking place on the milling ball surface or on abraded metal particles? We made several observations that support the milling ball hypothesis. For both, the Suzuki- and Glaser-coupling abraded metal powders showed little to no catalytic activity.^26,43^ Furthermore, reactions where the abrasion of the milling ball was near or below the detection limit still afforded quantitative yields. In order to investigate this further, we attempted to detect the copper acetylide intermediates of the Glaser coupling inside the bulk solid. However, we identified the presence of the investigated intermediate exclusively on the milling ball surface (Fig. 6). We thus conclude that the point of the catalysis is indeed the milling ball surface itself.

After having identified the location, it is likewise crucial to establish the point in time the reaction is occurring. Here the evidence is scarce and inconclusive. It seems reasonable that the moment of impact, *e.g.* ball–vessel collision, is also the point in time of the reaction, but there are some considerations that contradict this hypothesis. For example, a milling ball with a diameter of 10 mm possess an active surface of 3.14 × 10^14^ nm^2^. However, only a fraction of this surface is at the point of contact. Though, even if the entire surface were the place of a reaction and furthermore, rather big molecules are considered (*i.e*., 1 nm^2^ each) to be transferred at once, this would only amount to roughly one millionth of the total molecules present under typical reaction conditions. With perfect diffusion and complete replacement of the surface with unreacted reactants between each collision, a full conversion would require 1.000.000 collisions. At 30 Hz and the resulting 60 collisions per second for an idealized system with one milling ball, that would amount to roughly 4.6 hours of milling. In our experiments, however, we observed full conversion already after 30 minutes. One collision thus has to convert more molecules than adsorbed on a monolayer of the ball. In order to understand this, it is crucial to establish and simulate the processes at the point of impact. While some simulations show, that these events lead to an enhanced diffusion between solids, more simulations and tailored experiments with direct mechanocatalysis in mind are necessary to answer this question in the future. For now, we hypothesize that some explanation along the lines of the pseudo-fluid model^61^ with a significant increased diffusion towards and from the catalytically active centres enables the swift transformation of larger fractions powder within one conversion.

Another point of debate in this regard is the use of alloys. If one intentionally dilutes the active metal one would expect the activity to decrease alongside. For now, only substitutional copper alloys have been investigated, where copper is statistically replaced by tin or zinc in order to achieve a higher hardness. Since the distribution in the alloy is random, one can assume a reduction of catalytically active surface in the same amount as added inert material. For bronze this would amount to only 8%, while brass is already introducing 36% of inert material and in the case of K-Monel 70% of nickel are added.^43^ For the first two cases the activity even exceeded the pure copper in the Glaser coupling. More interestingly, for K-Monel, the yield was reduced by about 68% compared to brass. Thus, the introduction of inert material has an influence on the activity but more investigations with a systematic variation of active component is necessary to determine the magnitude and possible threshold values. Furthermore, one should try to eliminate other factors like, energy transfer, abrasion and hardness in order to draw unbiased conclusions.

In order to elucidate the reaction mechanism behind mechanochemical transformations, *in situ* investigations have proven an irreplaceable tool to overcome the black-box nature of the technology. While *ex situ* analysis can contribute (*i.e.*, stopping the reaction, opening the vessel, analysing the reaction temperature/progress, and then restarting the reaction), this procedure can influence the made observations and is thus less reliable and artefacts might be introduced. Herein, the reaction itself might continue even after the mill is stopped and, for instance, it has been shown that some reactions occur if a finely mixed powder is exposed to air. In the last years the development of *in situ* X-ray diffraction and Raman spectroscopy have helped to identify intermediates and aided in the establishment of putative mechanisms of mechanochemical transformations. In the case of DM, our group has utilized both techniques to follow the reaction progress. We could observe an induction period for both the Glaser coupling and the Suzuki coupling: in the first few minutes of the reaction, no product formation could be observed. There could be several reasons for this behaviour. One assumption is that the milling ball surface has to be covered with the reactants before the reaction can take place. This adsorption process however, would be way to long in combination of the achieved yields per collision as detailed above. Another cause might be the need of a fine intermixing of the reactants. Only if a certain distribution is achieved, the collisions become effective for the conversion. This also seems unlikely since in the case of the Glaser reaction, only one reactant is present. Yet another theory is that the reaction needs thermal energy to transpire. Due to the constant collisions, some energy is dissipated as heat. In our investigations inside vibratory ball mills with polymer vessels, however, the rise in temperature is in the order of 5 to 10 K. While it seems unlikely that such a small temperature increase can switch on a reaction, an in-depth study with temperature-controlled milling equipment can clear this up easily.

Altogether the mechanisms behind DM are not yet fully understood, but we can benefit directly from the research conducted for classical mechanochemistry and the equipment and techniques necessary are mostly available. Considering that only a handful of publications have been released on the topic, our understanding is already better than expected. We encourage the reader and interested colleagues to tackle the questions raised in this paragraph in order to further the understanding of this promising catalytic method.

## Conclusions

The synthesis concept of direct mechanocatalysis (DM) enables the conduction of catalytic reactions in ball mills without requiring the addition of a soluble catalyst or a catalytically active powder. In DM, the milling ball itself is the catalyst. Up to now, DM has been successfully employed for various coupling, hydrogenation, and cycloaddition reactions by using metal balls made from Cu, Ni, Pd, or steel. The catalysis mechanism of DM is largely unexplored, but is typically thought to notably differ from corresponding solution-based reactions. The range of further potential catalytic transformations is tremendous and we are still at the very beginning of a promising journey that is likely to yield a wealth of new synthetic options and improvements. This article provided a summary of the current state of the art as well as a guideline of the relevant underlying concepts, mechanistic considerations, and impactful parameters and milling additives that need to be considered in DM. We expect the field of direct mechanocatalysis to grow substantially over the upcoming years and we hope that this perspective may inspire young mechanochemists as well as classical organic chemists to adopt this facile synthesis concept in their future synthetic endeavours.

## Author contributions

S. H., S. G., and L. B. wrote the manuscript jointly.

## Conflicts of interest

There are no conflicts to declare.

## Supplementary Material
